# Editorial: Cardiomyopathies: Current Treatment and Future Options

**DOI:** 10.3390/jcm9113531

**Published:** 2020-10-31

**Authors:** Stefan Peters

**Affiliations:** Internal Medicine-Cardiology, Ubbo Emmius Hospital Norden, Cardiology, Osterstr. 110, 26506 Norden, Germany; stefan.peters@u-e-k.de; Tel.: +49-4931-181-435

**Keywords:** cardiomyopathy, hypertrophic, dilated, arrhythmogenic

## Abstract

Cardiomyopathies are an essential component in clinical cardiology. The number of different cardiomyopathies have increased a lot due to genetics and newer insights in pathomechanism. The current treatment and future options are demonstrated in advance.

Cardiomyopathies are an essential factor in cardiology with important progress in genetics, medical treatment and treatment with devices. Ablation in order to treat various forms of arrhythmias plays a more and more increased role.

In 1995, the most important cardiomyopathies were dilated cardiomyopathy, hypertrophic cardiomyopathy, restrictive cardiomyopathy and arrhythmogenic right ventricular cardiomyopathy [1].

A lot of other cardiomyopathies have been described in recent years, such as non-compaction cardiomyopathy, takotsubo cardiomyopathy, and many other subtypes of already known cardiomyopathies [2].

In hypertrophic cardiomyopathy, we must differentiate between various forms of hypertrophy. We can offer articles to various types of hypertrophy due to different causes like Amyloid cardiomyopathy and Fabry cardiomyopathy. The same genes for hypertrophic cardiomyopathy can cause non-compaction left ventricle, which is included in our reviews.

The definition of arrhythmogenic cardiomyopathies has changed in recent years; meanwhile, arrhythmogenic dilated cardiomyopathy, arrhythmogenic right ventricular cardiomyopathy, and arrhythmogenic left ventricular cardiomyopathy are included. What is more important is the fact that different genes play a crucial role, like Filamin C, Lamin A/C, phospholamban and RBM20. We could invite international experts in this field to write important papers.

The importance of ajmaline testing in arrhythmogenic right ventricular cardiomyopathy and hypertrophic cardiomyopathy is worth a particular paper in order to predict the risk of life-threatening ventricular arrhythmias.

Idiopathic dilated cardiomyopathy has gained intensive interest due to different stages of the disease. Arrhythmogenic dilated cardiomyopathy, non-dilated hypokinetic left ventricle, and dilatation without contraction impairment were described in 2016 [3]. The progress in definition are described by experts in the field.

Several cases of rare cardiomyopathies hide under the term of non-ischemic heart failure with preserved left ventricular ejection fraction and benefit from newer treatment options like tafamidis, and in the near future, mavacamten.

A lot has changed in the definition of cardiomyopathies since 1995, summarized in present papers with increased knowledge in genetics, pathophysiology, medical treatment, the use of devices, and ablation techniques, and future options in treatment are developing in the coming years.

## References

[1] Richardson P., McKenna W., Bristow M., Maisch B., Mautner B., O’Connell J., Olsen E., Thiene G., Goodwin J., Gyarfas I. (1996). Report of the 1995 World Health Organization/International Society and Federation of Cardiology Task Force on the definition and classification of cardiomyopathies. Circulation.

[2] Maron B.J., Towbin J.A., Zhiene G., Antzelewitch C., Corrado D., Arnett D., Moss A.J., Seidman C.E., Young J.B. (2006). Contemporary definitions and classification of cardiomyopathies. Circulation.

[3] Pinto Y.M., Elliott P.M., Arbustini E., Adler Y., Anastasakis A., Böhm M., Duboc D., Gimeno J., de Groote P., Imazio M. (2016). Proposal for a revised definition of dilated cardiomyopathy, hypokinetic non-dilated cardiomyopathy, and its implications for clinical practise: A position statement of the ESC working group on myocardial and pericardial diseases. Eur. Heart J..

